# The Ankyrin-Repeat Gene *GmANK114* Confers Drought and Salt Tolerance in *Arabidopsis* and Soybean

**DOI:** 10.3389/fpls.2020.584167

**Published:** 2020-10-29

**Authors:** Juan-Ying Zhao, Zhi-Wei Lu, Yue Sun, Zheng-Wu Fang, Jun Chen, Yong-Bin Zhou, Ming Chen, You-Zhi Ma, Zhao-Shi Xu, Dong-Hong Min

**Affiliations:** ^1^College of Agronomy, Northwest A&F University/State Key Laboratory of Crop Stress Biology for Arid Areas, Yangling, China; ^2^Institute of Crop Science, Chinese Academy of Agricultural Sciences (CAAS)/National Key Facility for Crop Gene Resources and Genetic Improvement, Key Laboratory of Biology and Genetic Improvement of Triticeae Crops, Ministry of Agriculture, Beijing, China; ^3^College of Agriculture, Yangtze University, Jingzhou, China

**Keywords:** ankyrin repeat protein, genome-wide analysis, responsive mechanism, drought and salt tolerance, soybean

## Abstract

Ankyrin repeat (ANK) proteins are essential in cell growth, development, and response to hormones and environmental stresses. In the present study, 226 *ANK* genes were identified and classified into nine subfamilies according to conserved domains in the soybean genome (*Glycine max* L.). Among them, the *GmANK114* was highly induced by drought, salt, and abscisic acid. The *GmANK114* encodes a protein that belongs to the ANK-RF subfamily containing a RING finger (RF) domain in addition to the ankyrin repeats. Heterologous overexpression of *GmANK114* in transgenic *Arabidopsis* improved the germination rate under drought and salt treatments compared to wild-type. Homologous overexpression of *GmANK114* improved the survival rate under drought and salt stresses in transgenic soybean hairy roots. In response to drought or salt stress, *GmANK114* overexpression in soybean hairy root showed higher proline and lower malondialdehyde contents, and lower H_2_O_2_ and O^2–^ contents compared control plants. Besides, *GmANK114* activated transcription of several abiotic stress-related genes, including *WRKY13*, *NAC11*, *DREB2*, *MYB84*, and *bZIP44* under drought and salt stresses in soybean. These results provide new insights for functional analysis of soybean ANK proteins and will be helpful for further understanding how ANK proteins in plants adapt to abiotic stress.

## Introduction

In recent years, various abiotic stresses, especially drought and salt, became more and more frequent with global climate change. Abiotic stresses change the physiological traits and metabolism of plants, affect the growth and development processes, and eventually lead to decreased yield and quality of crops (Veatch-Blohm, 2007; Hasanuzzaman et al., 2012). To adapt to the changing environments, plants induce the expression of stress-related genes by receiving external signals and undergoing a series of complex signal transduction pathways and diverse response mechanisms, thereby reducing the damage of abiotic stresses on their growth and development (Chen and Murata, 2011; Wang et al., 2015; Zhu, 2016; Qi et al., 2018; Yang and Guo, 2018).

The ankyrin repeat (ANK) proteins, with at least four consecutive ANK motifs (Michaely and Bennett, 1992; Mosavi et al., 2004), are distributed in diverse organisms ranging from viruses to plants (Sedgwick and Smerdon, 1999), and are very crucial in different functions, including cell cycle regulation, transcriptional regulation, cytoskeleton interaction, and signal transduction. In humans and animals, ANK proteins are directly involved in the development of cancer. The human *VBARP* is thought to be involved with cellular apoptosis (anti-apoptotic) and cell survival pathways (Miles et al., 2005).

Similarly, plant ANK proteins were involved in various important biological processes. AKR, the first reported ANK protein with five ankyrin repeats in *Arabidopsis*, played a regulatory role in cell differentiation and development (Zhang et al., 1992). EMB506, similar to AKR with five ankyrin repeats, is an essential chloroplast protein for normal development of *Arabidopsis* embryos (Albert et al., 1999). Subsequently, the EMB506 was proved interact with the AKR protein and caused to lose its function, thereby resulted in an embryo-defective phenotype. It was demonstrated that *EMB506* and *AKR* are involved in crucially and tightly controlled events in plastid differentiation linked to cell differentiation, morphogenesis, and organogenesis during the plant life cycle (Garcion et al., 2006). AtACD6, a regulator and an effector of salicylic acid (SA) signaling, is a dose-dependent activator of the defense responses against virulent bacteria and can activate SA-dependent cell death (Lu et al., 2003, 2005). The interaction of the tobacco ANK protein NEIP2 with ethylene receptor NTHK1 improved plant growth and performance under salt and oxidative stresses (Cao et al., 2015).

In previous studies, ANK proteins were divided into multiple subfamilies according to the conserved domains (Becerra et al., 2004). The ANK-RF subgroup proteins contain the RING finger (RF) domain, which was first identified in the *Really Interesting New Gene* (Freemont et al., 1991). The RF domain, belonging to the zinc finger domain protein family, could bind to RNA, protein or lipid substrates (Elenbaas et al., 1996). A large number of RF-containing proteins have an E3 ligase role in ubiquitination reactions (Stone et al., 2005; Berrocal-Lobo et al., 2010) and the C3HC4 motif of RF domain is essential for conferring E3 ligase activity (Lorick et al., 1999). Rice XA21-binding protein 3 (XB3), an ANK-RF E3 ubiquitin ligase, is a substrate for the XA21 kinase and interacts with the XA21 kinase domain for full accumulation of the XA21 protein and for *XA21*-mediated immunity. Therefore, XB3 contributes to the stability of XA21 and is required for full accumulation of the XA21 protein in *XA21*-mediated rice immunity (Wang et al., 2006).

ANK-RF proteins functions in various biological processes in plant growth and development. The *Arabidopsis* ANK-RF protein AtXBAT32, an ubiquitin ligase, positively affected ethylene biosynthesis by degradation of the ethylene biosynthetic enzyme1-aminocyclopropane-1-carboxylate synthase 7 (Lyzenga et al., 2012), resulting in regulation of lateral root initiation (Nodzon et al., 2004; Prasad et al., 2010). Another ANK-RF protein AtXBAT35 participated in ubiquitin-mediated protein degradation, and played an important role in negatively regulating ethylene-mediated apical hook curvature in *Arabidopsis* (Carvalho et al., 2012). The overexpression of *LIANK*, a lily ANK-RF protein, resulted in pollen tube growing abnormally and its silencing impaired pollen germination and tube growth (Huang et al., 2006).

Recently, ANK-RF subfamily genes were proven to play diverse roles in the stress responses. A RING zinc finger ankyrin protein gene *AdZFP1* was isolated from drought-tolerant *Artemisia desertorum* and the transcript level of *AdZFP1* was strongly induced by drought, salt, cold, heat, and exogenous abscisic acid (ABA) treatments. Besides, overexpression of the *AdZFP1* gene enhanced drought tolerance in transgenic tobacco (Yang et al., 2008). The pepper ANK-RF gene *CaKR1* participates in various biotic and abiotic stresses, such as NaCl, cold, SA, ethylene, and pathogens infection. Overexpression of *CaKR1* enhances resistance to salt and oxidative stresses in tomato (Seong et al., 2007a). The potato ANK-RF gene *Star*, a putative E3 ubiquitin ligase gene, is involved in late blight resistance and organ development (Wu et al., 2009). The rice ANK-RF protein *OsXB3* plays a role in resistance against *Xanthomonas oryzae pv. Oryzae* (Wang et al., 2006).

Soybean (*Glycine max* L.), an important crop providing plant protein and oil, suffers from drought and salt damage with global climate change, especially in China. Previous studies characterized the *ANK* gene families in various plants (Becerra et al., 2004; Huang et al., 2009; Jiang et al., 2013; Yuan et al., 2013; Lopez-Ortiz et al., 2020). In this study, we identified 226 non-redundant soybean *ANK* genes, which were classified into nine subfamilies. An ANK-RF subfamily gene *GmANK114* was significantly induced by drought, salt, and exogenous ABA. We further investigated stress tolerance conferred by *GmANK114* in both *Arabidopsis* and soybean. The functional identification of *GmANK114* will be very helpful for further understanding how ANK proteins in soybean adapt to abiotic stresses.

## Materials and Methods

### Identification of Soybean *ANK* Genes

We obtained the Hidden Markov Model (HMM) profile of the ANK domain (PF00023) from Pfam v29.0.^1^ BLAST was used to identify putative *GmANK* with the ANK domain (PF00023) as a query against the Phytozome database (v12.1)^2^ of soybean. All hits with expected values less than 1.0 were retrieved and redundant sequences were removed using BLASTclust.^3^ Then, all candidate sequences that met the standards were analyzed manually in the Pfam database once more and were checked using the SMART program (Letunic et al., 2009) for the purposes of eliminating any sequences not containing the ANK domain (Liu et al., 2016a). The ExPASy website was used to predict physio-chemical parameters of ANK proteins such as molecular weights (Mw) and theoretical isoelectric points (pI) (Artimo et al., 2012).

### Phylogenetic Relationships and Classification of GmANKs

The phylogenetic relationships among ANKs were inferred using Clustal X with a gap opening penalty of 10 and a gap extension penalty of 0.1 (Thompson et al., 1997). A phylogenetic tree was constructed using the neighbor-joining method in MEGA 7 and bootstrap analysis was conducted using 1,000 replications (Tamura et al., 2011). Database tools in SMART were used to analyze their typical functional structure domains. The sample protein structures of each subfamily were drawn manually.

### Gene Structure Prediction, Motif Analysis and Promoter Analysis

An exon-intron substructure map was produced by tools online GSDS 2.0 (Gene Structure Display Sever)^4^ (Guo et al., 2007). The conserved domain motifs analyze of soybean ANK proteins were conducted by TBtools (Chen et al., 2020), according to the analyzed result from MEME Suite 5.1.1.^5^ The motifs number were set for 10. The 2,000 bp region upstream of the initiation codon as the promoter region of the *GmANK-RFs* were selected to identify the *cis-*acting elements by submitting the promoter sequence to the PLACE^6^ and PlantCARE^7^ databases (Li et al., 2019).

### Physical Mapping and Gene Duplication

All non-redundant *ANKs* were mapped on the 20 chromosomes on the basis of information in the soybean database using the MG2C website (MapGene2Chrom web v2)^8^ (Liu et al., 2016b). Segmental and tandem duplication events were determined as previously described (Wang et al., 2016; Fan et al., 2019). Gene segmental duplications events were analyzed and visualized by Multiple Collinearity Scan toolkit (MCScanx)^9^ and Circos-0.67 program^10^ respectively, with a E-value for 10^–10^. The tandem duplications were characterized as adjacent genes with a distance of less than 200 kb within neighboring intergenic region (Holub, 2001). Tandem duplications were manually marked on the physical map.

### Gene Ontology Annotation

The functional annotation for the GmANK genes were conducted by Blast2GO^11^ software according to the previous description, including the subsequent analysis of annotation results (Conesa and Gotz, 2008).

### Expression Analysis of Soybean *ANK-RF* Genes

Transcriptome data was obtained from the Phytozome database to investigate the expression profile of 17 *ANK-RF* genes in different soybean tissues, including root, root hairs, stem, leaves, nodules, flower, and seed of soybean. These transcriptome data were obtained under normal growing conditions and were not subjected to any stress. RNA-seq data of various abiotic stresses were extracted from our previous research to study the expression of *ANK-RF* genes under drought, NaCl and ABA treatments (Shi et al., 2018). The RNA-seq data were obtained from three biological replicates. Tophat and cufflinks were used to analyze the RNA-seq expression, and the gene expressions were uniformed in fragments per kilobase million (FPKM). The expression of *ANK-RF* genes was extracted from the total expression data. The FPKM values of soybean *ANK-RF* genes in different tissues and under abiotic stress treatments (drought, NaCl and ABA) were shown in Supplementary Tables 2, 3. TBtools software was used to generate the heatmap (Chen et al., 2020).

### Plant Materials and Stresses Treatment

Seeds of soybean variety Williams 82 were grown in a greenhouse at 28/20°C day/night temperatures, with a photoperiod of 14 h light/10 h dark and 60% relative humidity. For drought treatment, 16-day-old seedlings were placed on filter paper for the induction of rapid drought for 0, 1, 2, 4, 8, 12, and 24 h. For NaCl treatment, 16-day-old seedlings in soil watered with 200 mM NaCl solution. For ABA treatments, the seedlings were exposed to 100 μM ABA. Unstressed plants were maintained as control. After stress treatments, the seedlings were carefully harvested and immediately frozen with liquid nitrogen, and stored at −80°C until RNA isolation.

Wild-type (WT) and T3 transgenic seeds were used to evaluate the drought and salt tolerance. For germination assay, seeds were cultured in the medium with 6 or 9% (w/v) PEG 6000 and 75, 100, and 125 mM NaCl. After 3 days of vernalization, the seeds were transferred to normal conditions for germination. Seeds were considered to be germinated when radicles emerged from the seed coats. The percentage of germinated seeds was calculated based on the number of seedlings at 1, 2, 3, 4, 5, and 6 day. All the experiments were repeated three times.

### *Agrobacterium rhizogenes*-Mediated Transformation of Soybean Hairy Roots

The open reading frame (ORF) of *GmANK114* was amplified and ligated into plant transformation vector pCAMBIA3301 driven by the CaMV35S promoter to generate the pCAMBIA3301-*GmANK114* overexpression vector. The recombinant vectors were introduced into *Agrobacterium rhizogenes* strain K599, and that was used to infect soybean cultivar Williams 82 cotyledonary node by injection as described previously (Kereszt et al., 2007). All experiments have three biological repetitions at least.

### Measurements of Proline, MDA, H_2_O_2_, and O^2–^ Contents

Before measurements, the transgenic soybean hairy roots were subjected to drought or 200 mM NaCl stress for 5 days, after which the proline (Pro), malondialdehyde (MDA), H_2_O_2_, and O^2–^ contents of leaves were measured using the corresponding assay kit (Solarbio, Beijing, China) in accordance with the manufacturer’s protocol. All measurements were repeated three times.

### RNA Extraction and Quantitative Real-Time PCR

Total RNA was isolated using Trizol reagent (TaKaRa, Japan) and treated with RNase-free DNase I (TaKaRa, Japan) to remove genomic DNA contamination. The cDNA synthesis and reverse transcription-PCR were conducted using TransScript All-in One First-Strand cDNA Synthesis SuperMix for qPCR (TRANSGEN, China). Quantitative real-time PCR (qRT-PCR) was performed in three technical replicates using Super Real PreMix Plus (SYBR Green) (TIANGEN, China) with an Applied Biosystems^®^ 7500 Real-Time PCR System. The soybean *CYP2* (Glyma.12g024700) gene was used for normalization (Zhang et al., 2019). The primers used for qRT-PCR are listed in Supplementary Table 4.

### DAB and NBT Staining

The detached leaves of transgenic soybean were stained separately after stress treatment. For 3,3-diaminobenzidine (DAB) and nitroblue tetrazolium (NBT) staining, the samples were immersed in DAB or NBT solution (Solarbio, China) for 12 h and then transferred to 75% ethanol for decoloring until the leaves became white (Du et al., 2018).

### Statistical Analysis

All experiments above were replicated three times independently. The values are shown as mean ± standard deviation (SD). ANOVA test was used for statistical analyses, and the signifcance was labeled ^∗^*P* < 0.05; and ^∗∗^*P* < 0.01.

## Results

### Identification of Soybean *ANKs*

The ANK domain (PF00023) was used as a query and searched in several databases including Phytozome, NCBI, Pfam, and SMART, a total of 226 genes encoding ANK proteins were identified in soybean. Almost all the *GmANKs* contained introns. The number of introns in these genes ranged from 0 (*GmANK9*, *GmANK12* and *GmANK14*) to 21 (*GmANK53*) (Supplementary Figure 1). These results suggested that members of the *GmANK* family might be active and constantly evolving.

The size and physicochemical properties of *GmANKs* varied substantially. The length of GmANK proteins ranged from 133 (GmANK7) to 1,637 (GmANK122) amino acids. The molecular weights of GmANK proteins changed from 14,747.23 Da (GmANK7) to 179,852.44 Da (GmANK122). The characteristic features of GmANK protein sequences are summarized in Supplementary Table 1.

### Chromosomal Location and Duplication Events Analysis

**FIGURE 1:**
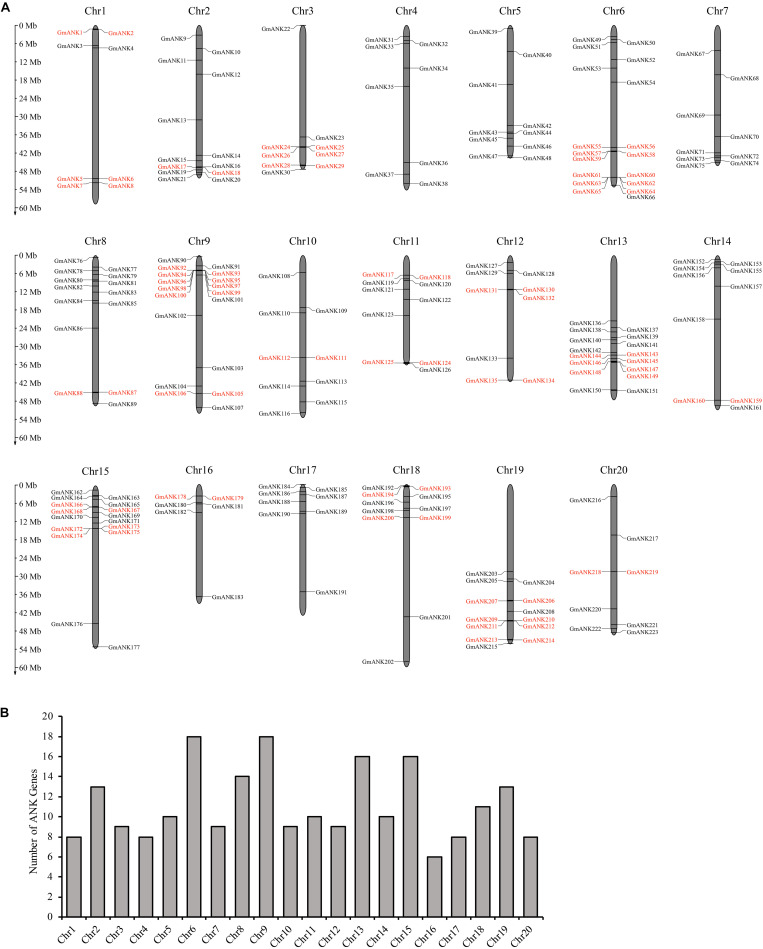
Distribution of 223 *GmANK* genes on the soybean chromosomes. **(A)** The physical location of each member in soybean. The scale on the left is in megabases (Mb). The chromosome number (Chr1–Chr20) is indicated at the top of each chromosome. The tandem duplicated gene clusters are marked by the red rectangle. **(B)** The numbers of *ANK* genes were distributed on 20 chromosomes.

To determine the genomic distribution of *ANK* genes, chromosomal localization maps were constructed in soybean. The precise position (in bp) of each *GmANK* gene on soybean chromosomes is detailed in Supplementary Table 1. In total, 223 of the 226 *GmANKs* on chromosomes revealed an uneven distribution in the genome. As shown in Figure 1, the number of genes per chromosome ranged from 6 to 18. Chromosomes 6 and 9 had the largest number of *GmANK* genes (18 members), followed by 16 *GmANK* genes on each of chromosomes 13 and 15, while low densities of *GmANK* genes were observed in chromosome 16 (six members).

**FIGURE 2:**
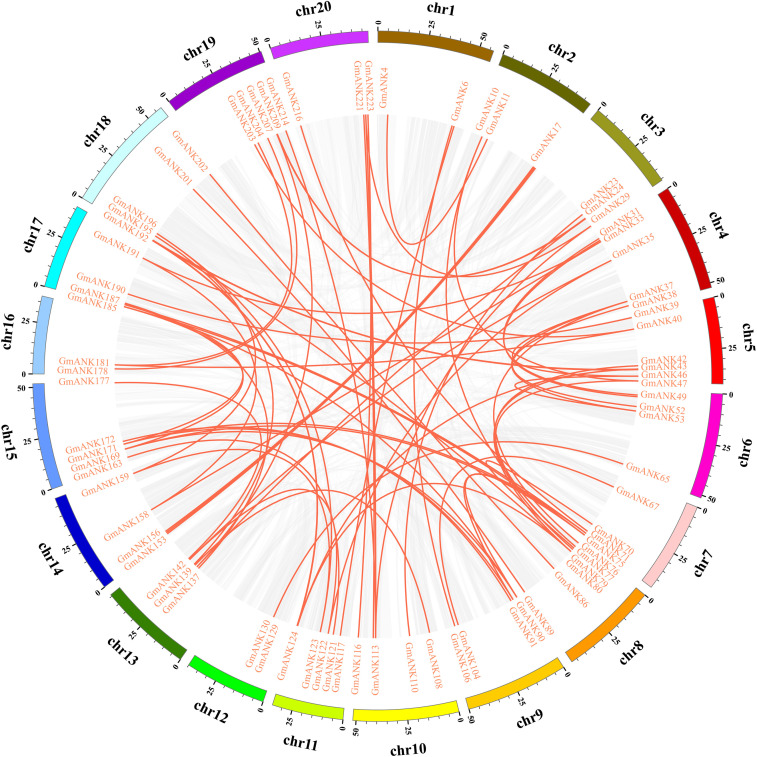
Distribution of segmentally duplication *ANK* genes on soybean chromosomes. Gray lines indicate all synteny blocks in whole soybean genome, and red lines indicate duplicated *ANK* gene pairs.

To elucidate the conceivable mechanism of evolution of *GmANKs*, tandem and segmental duplication events were analyzed. A total of 81 *GmANK* genes were involved in tandem duplications consisting of 34 clusters (Figure 1). The distance between these genes ranged from 2.8 to 180.4 kb and five tandemly duplicated *GmANK* sets contained more than three genes. Among them, two tandemly duplicated *GmANK* sets contained three members (*GmANK57/58/59*, *GmANK166/167/168*), one tandemly duplicated *GmANK* set contained four members (*GmANK172/173/174/175*), one tandemly duplicated *GmANK* set contained six members (*GmANK57/58/59/166/167/168*), the maximum tandemly duplicated set contained nine members (*GmANK92/93/94/95/96/97/98/99/100*) and the other tandem duplications contained only two genes. Besides tandem duplication events, we further observed that up to 46% (104 out of 226) of *GmANK* genes were involved in segmental duplication and 36 genes were duplicated more than twice (Figure 2). In general, these results show that tandem and segmental duplication may be one of the main contributing factors for the large expansion of *ANK* genes in the soybean genome.

### Classification of GmANKs

**FIGURE 3:**
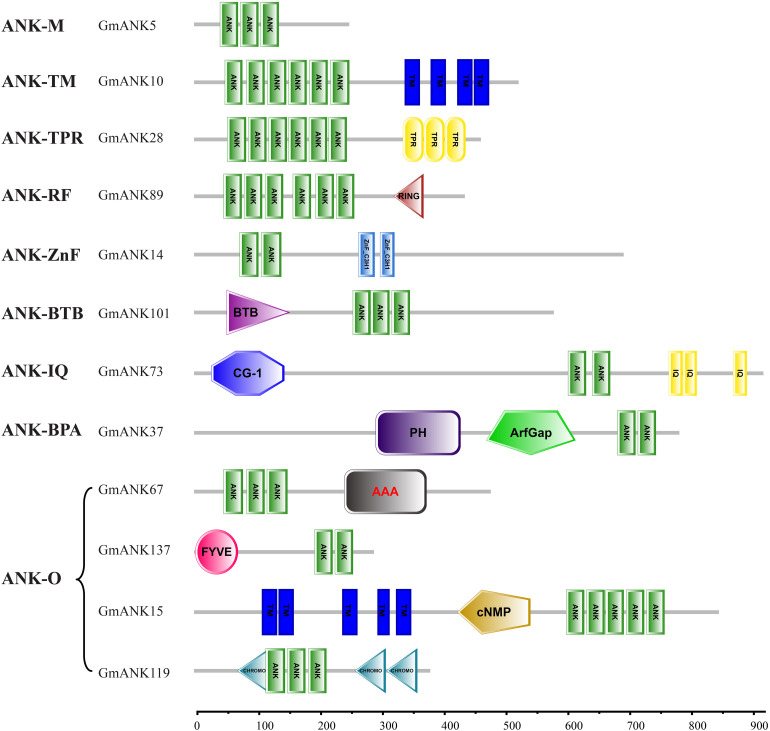
Domain compositions of representative GmANK proteins from each subfamily. The subfamily name of each corresponding protein and gene ID of a representative protein of the family are given on the left. Different domains are indicated with different colors and abbreviations. The length, order and number of domains represent the actual situation in each protein. Domain abbreviations are: ANK, ankyrin repeat domain; TM, transmembrane; TPR, tetratricopeptide repeat domain; RING, ring finger domain; ZnF-C3H1, zinc finger; BTB, BTB/POZ domain; CG-1, calmodulin-binding transcription activator; IQ, calmodulin-binding domain; PH, pleckstrin homology domain; ArfGap, putative GTP-ase activating proteins for the small GTPase; AAA, ATPase family associated with various cellular activities; FYVE, FYVE zinc finger; cNMP, cyclic nucleotide-binding domain; CHROMO, chromatin organization modifier domain.

Based on the domain compositions (Huang et al., 2009), the 226 GmANK proteins were classified into nine subfamilies according to the results of SMART searches (Figure 3). In total, 78 members that contained only one ANK domain belonged to subfamily ANK-M, while the remaining 148 proteins contained additional typical domains. Transmembrane domains were found in 78 GmANKs, which were identified as the ANK-TM subfamily. Seventeen members were confirmed as subfamily ANK-RF (ring finger). ANK-BTB subfamily (four members) had broad-complex, tramtrack and bric a brac domains. The other subfamilies included seven members with BAR, PH and ArfGap domains (ANK-BPA subfamily), two with tetratricopeptide repeat domains (ANK-TPR subfamily), 13 with zinc-finger domain (ANK-ZnF), and 11 contained conserved CG-1 (Calmodulin-binding Transcription Activator) and IQ (Calmodulin-binding motif) domains (ANK-IQ subfamily). ANK-O subfamily (16 members) contained other domains including FYVE, AAA, cNMP and CHROMO (Table 1).

**TABLE 1:** Number of *ANK* genes in different subgroups classified by the domains that they contained.

Subfamily	Detail information	Soybean	Maize	Rice	*Arabidopsis*	Tomato
ANK-M	Proteins with only ankyrin repeat	78	30	73	18	26
ANK-TM	Ankyrin-transmembrane proteins	78	15	37	40	25
ANK-TPR	Protein with tetratricopeptide repeats	2	2	22	1	4
ANK-RF	Proteins with Ring finger proteins	17	9	9	5	7
ANK-ZnF	Proteins with Zinc-finger proteins	13	3	7	6	8
ANK-BTB	Proteins with BTB domain	4	2	6	7	7
ANK-IQ	Proteins with calmodulin biding motif-containing protein	11	1	4	4	7
ANK-PK	Proteins with protein kinases	-	4	4	7	9
ANK-BPA	Proteins with BAR, PH and ArfGTPase-activating domain	7	2	3	4	4
ANK-IT	Protein with K^+^ channel protein	-	-	-	6	7
ANK-GPCR	Protein with GPCR-chaperone1 domain	-	-	-	-	4
ANK-MS	Protein with motile-sperm domain	-	-	1	-	4
ANK-O	Proteins with other domains	16	3	9	7	15
Total		226	71	175	105	130

### Phylogenetic Analysis of GmANK Proteins and Conserved Motif Analysis

**FIGURE 4:**
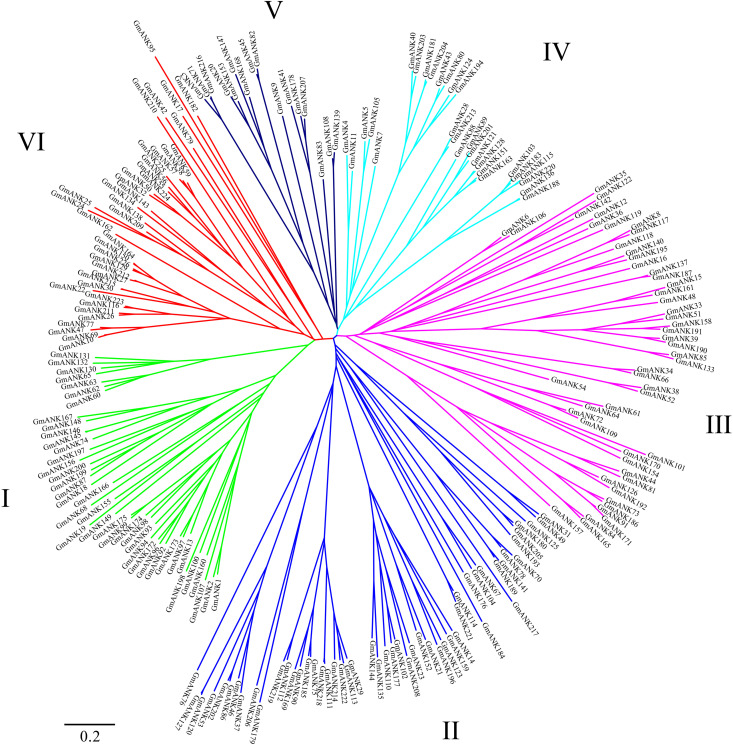
Phylogenetic analyses of ANK proteins in soybean. The complete amino acid sequences of the 226 GmANK proteins were aligned via Clustal X and were manually corrected. The phylogenetic tree was constructed with MEGA 7 in conjunction with the neighbor-joining method. This tree is drawn to scale with branch lengths in the same units as those of the evolutionary distances used to infer the phylogenetic tree. Six discrete groups (Cluster I–VI) were highlighted in different colors.

To understand the evolutionary relationships of GmANKs, an unrooted phylogenetic tree was constructed using the neighbor-joining method. The phylogenetic analysis categorized all GmANKs into six discrete groups (Group I–VI) comprising 41, 52, 50, 28, 16, and 39 proteins, respectively (Figure 4). Classification based on the phylogenetic tree and domain composition were consistent in general but not quite, such as for GmANK122 and GmANK142. Phylogenetic analysis reveals well-supported bootstrap values between GmANK122 and GmANK142 (Group III). However, GmANK122 has a RING domain (ANK-RF) but GmANK142 belongs to the ANK-M subfamily.

To better understand the function of each GmANKs subgroups, the motifs analysis of GmANKs family were conducted using MEME and TBtools software with default parameters (Supplementary Figure 2). The sizes of these conserved motifs ranged from 15 to 50 amino acids (Table 2). In general, the motifs structures in each subgroup of GmANK family were highly consistent and different combination of motifs represented different subgroups of GmANK family according to the analysis results of Supplementary Figure 2, which may account for the special biological functions of each groups and further confirmed their phylogenetic relationships.

**TABLE 2:** List of the identified motifs of GmANK proteins.

motifs	BEST POSSIBLE MATCH	width
1	IRDSDGRTPLHJAARRGHVEV	21
2	KNTANSCTVVATLIATVAFAAAFTVPGGV	29
3	GNTALHLAVRKGHVEVVKLLL	21
4	FRMYSFKVRPCSRAYSHDWTECPFVHPGENARRRDPRKYHYSCVPCPEFR	50
5	LFISLAVVVVQTSVVVIETKAKKQVVAVINKLMWLACVCIS	41
6	MFLSILTSRYAEEDFLKSLPLKLJFGLVTLFISIASMMVAF	41
7	ISGAALQMQWELKWFEEVKKJMPPSFIERKNSDGKTAREJFTEEHKELLK	50
8	TPLHVAAANGHVEVV	15
9	ELKQTVSDIKHEVHSQLEQTRQTRKRVQGIAKEJKKLHREG	41
10	GRTALHVAVRGGHSSVVKLJL	15

### Gene Ontology Annotation

To better understand the possible biological function of *GmANK* genes, Blast2GO software were used to performed the Gene Ontology (GO) annotation (Supplementary Figure 3). According to the GO annotation results, there were a total of 62 GmANK proteins were annotated in various biological processes, including signal transduction, regulation of membrane potential, positive regulation of transcription from RNA polymerase II promoter, regulation of proteolysis and S-adenosylmethionine metabolic process. There were 59 GmANK proteins were predicted to be related to molecular functions, such as transcriptional activator activity, RNA polymerase II core promoter proximal region sequence-specific binding, voltage-gated potassium channel activity, ubiquitin protein ligase binding, zinc ion binding and metal ion binding. Finally, 138 GmANK proteins were belonged to cellular components, which were located to membrane, integral component of membrane, cytoplasm, integral component of plasma membrane and ubiquitin ligase complex respectively. With the help of GO analysis results, we could primarily confirm the functional characteristics of GmANK proteins, like biological processes, molecular functions and cellular components, and will be very useful for its future functional researches in soybean.

### *GmANK-RFs* Promoters Contain Various Stress-Responsive Elements

Promoter sequences were analyzed by PlantCARE and some *cis*-acting elements were revealed in all *ANK-RFs* promotor regions, such as ABRE (response to ABA), MYB (response to drought), MYB-like (response to drought), MBS (involvement in drought-inducibility), MYC (response to drought and salt) and LTR (response to low-temperature). In addition, an auxin-responsive element (TGA-element) and a MeJA-responsive element (CGTCA-motif and TGACG-motif) were also identified (Table 3). This result indicated that the *GmANK-RFs* genes may be involved in abiotic stresses, especially in drought.

**TABLE 3:** cis-acting elements of *GmANK-RFs* promoter region in soybean.

	Gene Name	ABRE	MYB	MYB-like	MBS	MYC	LTR	CGTCA -motif	TGACG -motif	TCA -element	TGA -element
1	GmANK35	3	5	1		2	2	1	1	1	1
2	GmANK88	3	7	2	3	7	1	1	1		
3	GmANK89	1	1			3	3	2	2		1
4	GmANK103	1	3	1		7		2	2	1	2
5	GmANK114	1	6	2		4		1	1		
6	GmANK115	2	6	3		6	1				
7	GmANK121	4	2		2	4					
8	GmANK122	4	3	2		4	1				1
9	GmANK128	5	1			5		4	4		
10	GmANK136	2	10	2	1	5	2	2	2	1	1
11	GmANK151	1	4	2	1	6		1	1	2	1
12	GmANK163		2			5	1			3	
13	GmANK183	2	5	3		3		4	4	1	4
14	GmANK188	4	6	1	1	8	1				1
15	GmANK201	2	2		1	5	3	3	3		1
16	GmANK220	1	4	1		5	1	1	1		
17	GmANK221	5	4	1		6	1	1	1		

### Tissue-Specific Expression Patterns of *GmANK-RF* Genes

**FIGURE 5:**
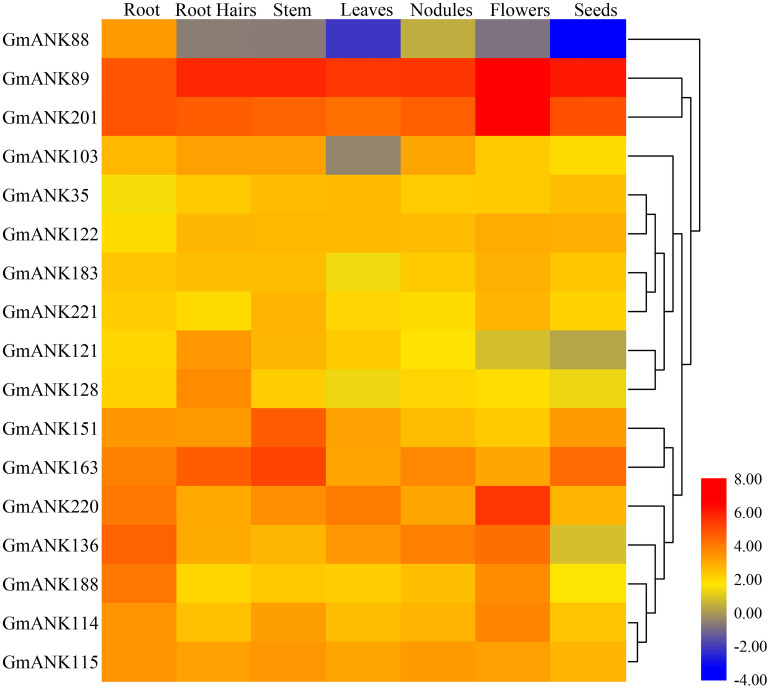
Heat map of expression profiles (in log2-based FPKM) of soybean *ANK-RF* subgroup genes in different tissues (root, root hairs, stem, leaf, nodules, flower and seed). The gene names are on the left and the tissue names are on the top of the figure, and the expression abundance of each transcript is represented by the color bar: Red, higher expression; blue, lower expression.

The expression abundance among different tissues and development stages may show diversity of different genes to adapt to various biological processes. To gain insights into the expression of *ANK-RF* genes in seven soybean tissues and organs, gene chip data were downloaded using publicly available RNA-seq data from the soybean genome database (Supplementary Table 2). The heatmap showed that 17 members of the ANK-RF subfamily were expressed in root, root hairs, stem, leaves, nodules, flowers and seeds (Figure 5). Among these *ANK-RF* genes, *GmANK89* and *GmANK201* had the highest expression in all tissues and organs, while *GmANK88* was only weakly expressed in root and *GmANK121* was slightly expressed in root hairs. Additionally, the expression patterns were different between *ANK-RF* genes in the same tissues. For example, the expression of *GmANK151* and *GmANK63* were at the highest level in stem, but *GmANK220* was expressed most strongly in flowers; *GmANK136* transcription had higher enrichment in root and *GmANK163* had stronger accumulation in root hairs. These transcriptional patterns suggested that the expression of these genes might be governed by diverse and potentially tissue-dependent regulatory mechanisms. *GmANK89* and *GmANK201* had extremely high expression in all tissues indicating that they may play specific roles in growth and development of soybean.

### *GmANK-RF* Genes Were Involved in Various Abiotic Stresses

**FIGURE 6:**
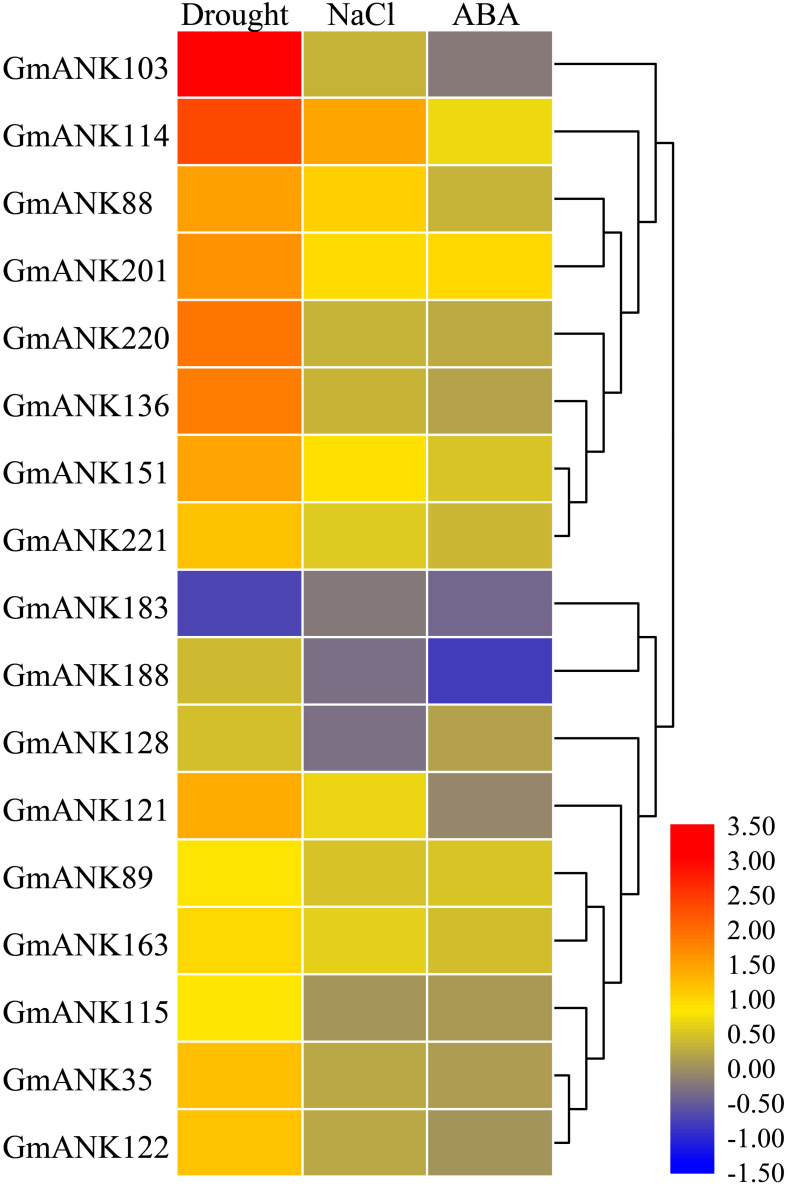
Transcriptome analysis-based soybean RNA sequencing data under drought, NaCl, and ABA. The expression abundance of each transcript is represented by the color bar: Red, higher expression; blue, lower expression.

Extensive studies have shown that ANK-RF subfamily genes may be involved in responding to various abiotic stresses in some species (Nodzon et al., 2004; Seong et al., 2007a, b; Yang et al., 2008). According to our previous research, RNA-seq was performed using soybean seedlings under drought, NaCl and ABA treatments (Shi et al., 2018). RNA-seq data indicated that 17 *GmANK-RFs* responded differently to drought, NaCl, and ABA treatments (Figure 6). Among them, several genes induced expressed under drought stress, such as *GmANK103*, *GmANK114*, *GmANK136*, and *GmANK220*. *GmANK114* were also specifically induced by NaCl. Under ABA stress, *GmANK183* and *GmANK188* showed down-regulated expression.

**FIGURE 7:**
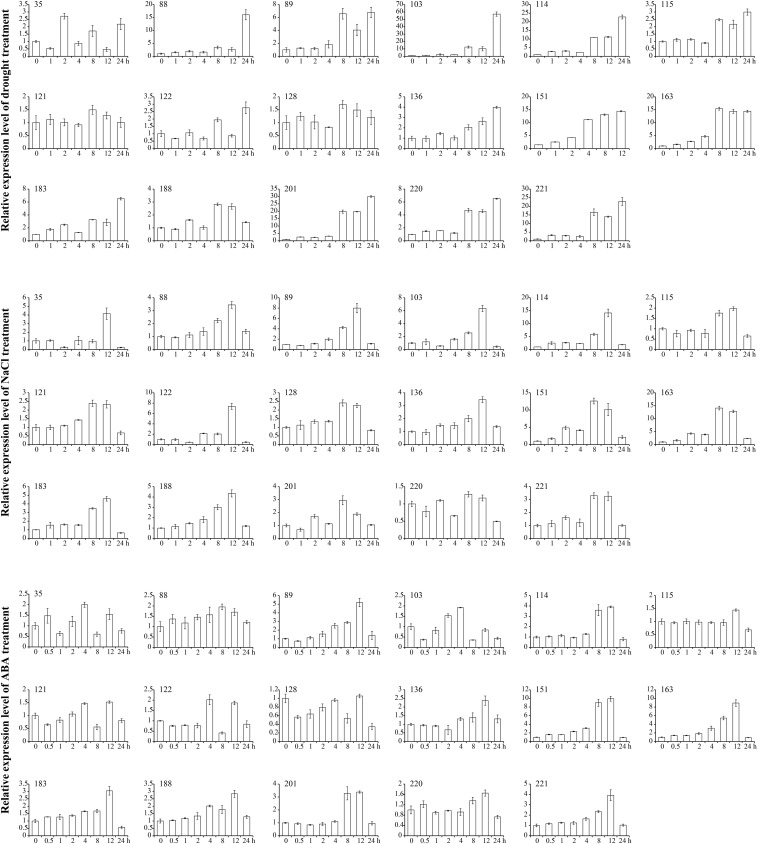
qRT-PCR analysis of 17 soybean *ANK-RF* genes under drought, NaCl and ABA treatments. The expression levels were normalized to that of *CYP2*. The mean and SD calculated from three biological replicates.

Based on the results of RNA-seq, we examined the expression patterns of 17 *GmANK-RF* genes during various abiotic stresses and phytohormone treatments by qRT-PCR (Figure 7). Quantitative real-time PCR results were roughly consistent with RNA-seq data. The expression levels of candidate genes varied in response to drought, NaCl, and ABA for 1, 2, 4, 8, 12, 24 h compared to untreated control samples. Under drought treatment, the expression of all *GmANK-RF* genes was increased, especially *GmANK103*, *GmANK114*, *GmANK201*, and *GmANK221*, which were induced more than 30-fold. For salt treatment, the peaks of *GmANK114*, *GmANK151*, and *GmANK163* transcription were increased to 15-fold. *GmANK89*, *GmANK114*, *GmANK151*, *GmANK163*, and *GmANK221* were also induced by ABA, however, the level of *GmANK115*, *GmANK121*, *GmANK128*, and *GmANK220* barely changed compared with 0 h. Through our transcriptional analysis of *GmANK-RF* genes, we found that the expression of *GmANK114* was significantly up-regulated under drought, salt, and ABA stress conditions (Figure 7). Therefore, *GmANK114* was selected for further verification.

### *GmANK114* Conferred Drought and Salt Tolerance in *Arabidopsis*

**FIGURE 8:**
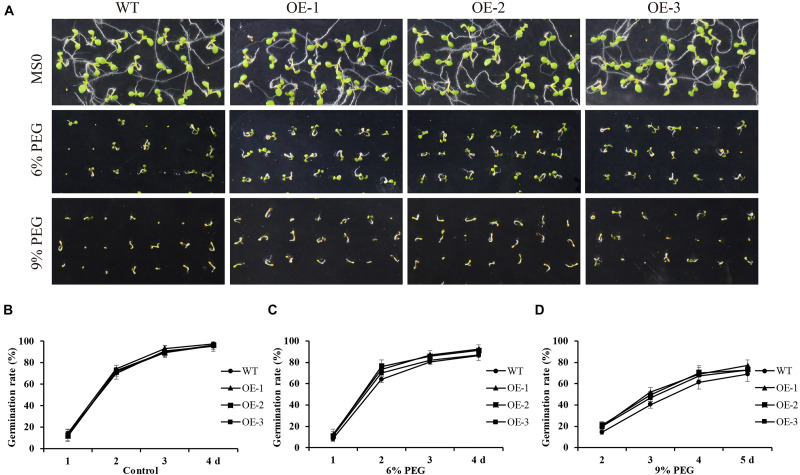
Overexpression of *GmANK114* enhanced seed germination rates under PEG6000 treatment. **(A)** Seed germination analysis of different lines seeds under 6% and 9% PEG6000 to simulated drought treatment. **(B–D)** Seed germination rates of WT and *GmANK114* transgenic *Arabidopsis* seeds at different time points. Date for each time point are means of three independent replicates.

*GmANK114* under the control of CaMV35S were transformed into *Arabidopsis* plants and T3 transgenic seeds were selected for tolerance identification. For drought treatment, the transgenic and WT seeds were germinated on Murashige and Skoog (MS) medium containing various concentrations of PEG6000 (Figure 8A). The transgenic and WT plants had no significant differences under the standard conditions in growth and morphology (Figure 8B), suggesting that over-expression of *GmANK114* genes might not affect plant growth and development under normal conditions. However, the germination percentage of transgenic plants was higher than the wild type in the presence of 6 and 9% PEG (Figures 8C,D). In growth medium with 9% PEG, 51.7, 48.9, and 46.2% of seeds germinated from three transgenic lines whereas only 40.1% of wild type germinated within 3 days (Figure 8D).

**FIGURE 9:**
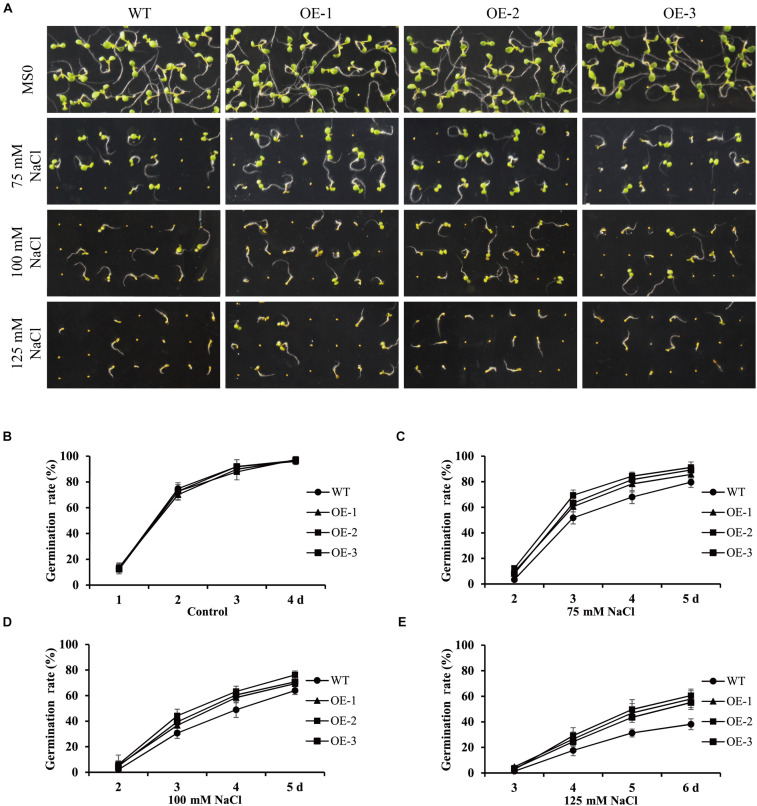
Germination rates of seeds in the presence or absence of NaCl in transgenic *Arabidopsis*. Values are means from three experiments. **(A)** Seed germination analysis of different lines seeds under 75, 100 and 125 mM NaCl to simulated salt treatment. **(B–E)** Seed germination rates of WT and *GmANK114* transgenic *Arabidopsis* seeds at different time points. Date for each time point are means of three independent replicates. The error bars indicate SD.

To further investigate the role of *GmANK114* under high salt, we observed the germination of overexpression *GmANK114* transgenic *Arabidopsis* seeds grown on medium containing various salt concentrations. For the germination assay, seeds of transgenic lines and WT were germinated on 75, 100, and 125 mM NaCl (Figure 9A), and the germination rates of both WT and transgenic seeds were determined. There was no difference in germination rate between transgenic lines and WT seeds in MS medium (Figure 9B), but the germination time and the germination rate in transgenic lines was earlier and higher than that in WT when treated with NaCl, especially for 125 mM NaCl (Figures 9C–E).

### *GmANK114* Improved Drought and Salt Tolerance in Transgenic Soybean Hairy Roots

**FIGURE 10:**
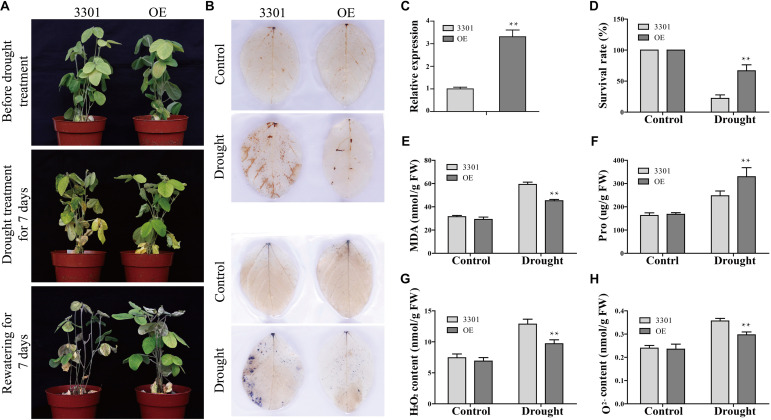
*GmANK114* improves drought stress tolerance in transgenic soybean hairy roots. **(A)** Phenotypes of *GmANK114*-overexpression and 3301-control transgenic soybean plants under before and after drought treatment. **(B)** DAB (top) and NBT (bottom) staining of OE and 3301 plant leaves under drought treatments. **(C)** Relative *GmANK114* expression in hairy roots of overexpressing *GmANK114* and control as shown by qRT-PCR. **(D)** Survival rate of normal and drought-stressed plants. **(E)** MDA and **(F)** proline content were detected in OE and 3301 plants under drought or normal growth condition. **(G)**The content of H_2_O_2_ and **(H)** O^2–^ in the leaves of *GmANK114*-OE and 3301-control plants after drought or normal condition for one week. The data were means ± SDs of three experiments. ANOVA test demonstrated that OE were significant differences (*P < 0.05 and **P < 0.01) compared with the corresponding controls.

**FIGURE 11:**
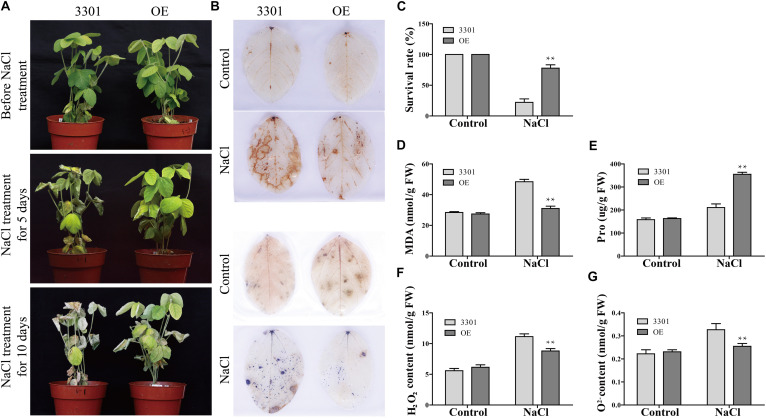
Phenotype and physiological indexes analysis of *GmANK114* transgenic soybean hairy roots under salt stress. **(A)** Phenotypes was evaluated in transgenic soybean plants after NaCl treatment. **(B)** DAB (top) and NBT (bottom) staining of leaves of OE and 3301 plants under NaCl treatments. **(C)** Survival rate of soybean hairy roots grown with salt treatment. **(D–G)** MDA, proline, H_2_O_2_ and O^2–^ contents in leaves of plants with transgenic hairy roots were measured under salt treatment for 7 days. All values are represented means for three biological replicates (*n* = 18). The error bars indicate SD. Significant differences (*P < 0.05 and **P < 0.01) are indicated by asterisks above the columns.

The function of *GmANK114* in drought and salt tolerance was further investigated by performing similar abiotic stress assays in *Agrobacterium rhizogenes*-mediated soybean hairy roots. Quantitative real-time PCR analysis showed that the expression level of *GmANK114* was significantly higher in hairy roots overexpressing *GmANK114* (OE) compared with the empty vector (3301) control (Figure 10C). For drought treatment, all hairy root seedlings including control grew in soil with no water for 2 weeks, and then rewatered for one week. Morphologically, no significant differences were observed for all experimental plants under normal growth conditions. Drought treatment caused obvious differences in growth and physiology between *GmANK114* overexpression lines and 3301-control. The leaves of all hairy root plants gradually yellowed and wilted with the drought treatment going on, but the control was more sensitive compared with the plants carrying the *GmANK114* under drought treatment after 7 days (Figure 10A). After rewatered for 7 days, 66.7% of the transgenic plants were still alive, while the control plants had a survival rate of only 22.2% (Figure 10D). The same phenomenon occurred with NaCl treatment and the survival rate in transgenic hairy roots was higher than that in control (Figures 11A,C).

In order to analyze the mechanism in improving drought resistance of *GmANK114*, MDA and Pro contents were determined under normal growth and stress conditions. Under normal growth conditions, the MDA content, which were 31.5 and 29.2 nmol/g in transgenic lines and 3301, respectively, did not differ among all plants. While under drought conditions, the MDA content in transgenic soybean was 45.2 nmol/g, significantly lower than that in control (59.3 nmol/g) (Figure 10E). The same results were obtained with salt treatment: the MDA contents in transgenic hairy root and CK were 30.9 and 48.4 nmol/g, respectively (Figure 11D). Under both drought and salt treatments, the Pro content in transgenic lines was higher than that in CK. By contrast, there was no significant difference in the Pro contents between transgenic lines and control under normal condition (Figures 10F, 11E).

Abiotic stress leads to the accumulation of reactive oxygen species (ROS), which can affect plant growth and development. Soybean leaves were stained with DAB and NBT to detect hydrogen peroxide (H_2_O_2_) and superoxide (O^2–^) contents in 3301 and OE. Under normal growth condition, no significant difference was observed in all plant leaves between the 3301-control and *GmANK114*-OE. However, the color depth of the transgenic hairy root leaves was significantly lower than that of the 3301 plants under drought and salt treatments (Figures 10B, 11B). The contents of O^2–^ and H_2_O_2_ in leaves were also measured and the results were consistent with the staining of DAB and NBT. Meanwhile, we found that the leaves of overexpressed *GmANK114* plants produced lower levels of free oxygen radicals with drought and NaCl treatments (Figures 10G,H, 11F,G). These results indicated that *GmANK114* positively regulated tolerance to drought and salt stresses in transgenic soybean hairy roots.

### GmANK114 Activated Transcripts of Stress-Responsive Genes in Soybean

**FIGURE 12:**
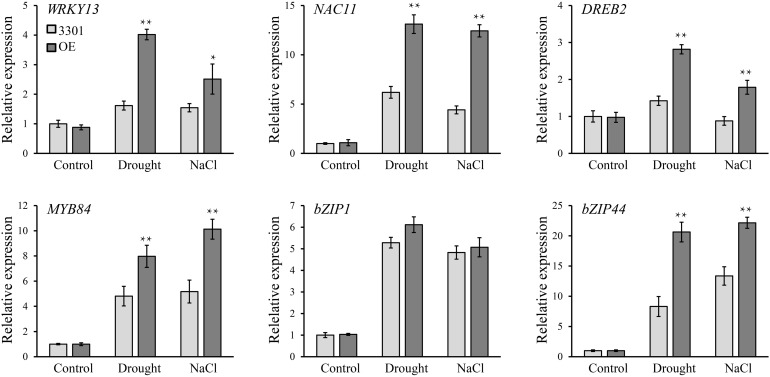
The relative transcript levels of *WRKY13*, *NAC11*, *DREB3*, *MYB84*, *bZIP1* and *bZIP44* in transgenic *GmANK114* and 3301 soybeans. qRT-PCR was used to detect expression levels under normal and stress treatment and *GmCYP2* was used as an internal control. Values are means and SD obtained from three biological replicates. The asterisks indicate a statistical significance (*P < 0.05 and **P < 0.01) compared with the corresponding controls.

To investigate the possible tolerance mechanisms, several drought- and salt-related genes were analyzed in transgenic soybean hairy roots under normal and stress conditions (Figure 12). When grown in normal conditions, we found that there were no significant differences in the expression level of these genes between 3301 and *GmANK114* OE lines by qRT-PCR. Under drought condition, the expression of *bZIP1* (Gao et al., 2011) had no clear difference, whereas transcript levels of the other genes in OE lines were higher than that in the 3301 control, including *WRKY13* (Zhou et al., 2008), *NAC11* (Hao et al., 2011), *DREB2* (Chen et al., 2007), *MYB84* (Wang et al., 2017), and *bZIP44* (Liao et al., 2008). Similarly, overexpression of *GmANK114* regulated transcripts of *WRKY13*, *NAC11*, *DREB2*, *MYB84*, and *bZIP44* under salt stress. These results indicated that *GmANK114* may be a key signaling molecule regulating plant drought and salt responses.

## Discussion

Drought and salt are the important factors that seriously affect the production and quality for soybean, especially for China which possess a population base. The ANK protein containing several conserved ankyrin repeats (2 to over 20) are important connexins protein that links membrane proteins and signaling molecules to the components of the underlying cytoskeleton (Li and Chye, 2004; Voronin and Kiseleva, 2008; Cunha and Mohler, 2009). ANK protein plays a key role in various cellular processes, participates in plant growth, development, intracellular protein transport, signal transduction, and stress responses (Wu et al., 2009; Brestic et al., 2015; Popescu et al., 2017). The study on the function of ANK protein will help to elucidate the mechanism that plants respond to environmental stresses, and provide the theoretical basis for environmental adaptation in plants.

Recently, the number of ANK proteins have been found is 105, 175, 130, and 71 in *Arabidopsis*, rice, tomato, and maize, respectively (Becerra et al., 2004; Huang et al., 2009; Jiang et al., 2013; Yuan et al., 2013). Although, it has been previously reported that 162 ankyrin repeats genes were found in soybean (Zhang et al., 2016), we identified 226 *ANK* genes in soybean and classified them into nine subfamilies. This difference maybe resulted from constantly updated in soybean genome database in recent years. The same subfamilies were found in *Arabidopsis*, rice, maize and soybean based on the domain compositions (Table 1), we inferred that these kinds of subfamilies were relatively conserved and they will take the similar functions in plants. The ANK-GPCR subfamily (protein with GPCR-chaperone1 domain) only found in tomato, but not in *Arabidopsis*, rice, maize and soybeans. Due to the differences in morphological structure, lifestyle and growth environment of plants evolution, some conserved subfamily genes have been retained to exercise the common physiological functions of plants, and specific genes have been developed to meet their own functional needs. ANK-GPCR subfamily genes may perform specific functions in tomato.

Several *ANK* genes may participate in responses to biotic and abiotic stresses. The expression of tobacco *NEIP2* was induced in the first few hours by treatment with salt and ethylene and further enhances plant performance under salt and oxidative stresses (Cao et al., 2015). ANK domain are crucial for YrU1 resistance to stripe rust in wheat (Wang et al., 2020). Based on this evidence, it was speculated that the soybean *ANK* genes probably involved in biotic and abiotic stress responses.

When plants are subjected to environmental stress, the second messenger perceives the stimulus in the environment and then modulates intracellular Ca^2+^ levels. The change of Ca^2+^ concentration often initiates a protein phosphorylation cascade that finally transfers to transcription factors controlling specific stress-regulated genes (Xiong et al., 2002). Transcription factors regulate the expression of target genes by binding to downstream gene promoters, thereby enabling plants to adapt to changes in the external environment. *ANK-RFs* were previously reported to be related to pathogen defense, plant growth and development, such as *XA21* and *XB3* (Wang et al., 2006). In this study, multiple *cis*-elements were found in the promoter region of *GmANK-RF* genes, including MeJA-responsive element (CGTCA-motif and TGACG-motif), which showed that *GmANK-RF* genes could function in defense responses. In addition, a number of stress response elements were also identified in the *ANK-RF* genes promoter region, such as drought response element MYB, MYB-like, MBS, salt response element MYC, low temperature element LTR, and ABA response element ABRE (Table 3). This indicates that *GmANK-RFs* might participate in multiple abiotic and biotic stress responses.

In the present study, the soybean *ANK-RF* gene *GmANK114* was shown to improve drought and salt tolerance in plants (Figures 10, 11). Proline functions in lowering the cellular osmotic potential and restoring intracellular solute concentrations, which prevents water loss from cells (Cui et al., 2018). MDA can inhibit the activity of cellular protective enzymes and reduce antioxidant content (Del Rio et al., 2005). When the enzyme and membrane system of plant tissues are destroyed, MDA content will be greatly increased (Tsikas, 2017). Therefore, MDA reflects the antioxidant capacity of plant tissues and can also be used as an indicator to measure the resistance of plants against external stress (Yang et al., 2019). Under drought and salt stresses, the differences of Pro and MDA contents between transgenic lines and controls showed that *GmANK114* could confer tolerance of soybean to drought and salinity. The intracellular ROS content affects plant growth and development. H_2_O_2_ and O^2–^ are the main sources of ROS, and can lead to the oxidative destruction of cells (Mittler et al., 2004). The contents of H_2_O_2_ and O^2–^ in leaves of transgenic soybean were significantly lower than that in control. This indicates that *GmANK114* gives soybean the ability to remove ROS. These results show that the *GmANK114* gene is involved in abiotic stresses and can scavenge ROS.

Plants have developed flexibly molecular and cellular mechanisms to fight against various abiotic stresses. *GmANK114* was induced by drought, NaCl, and ABA. To further explain the molecular mechanism of *GmANK114* participating in abiotic stresses, the expression levels of drought- and salt-related marker genes were tested by qRT-PCR. Transcription factor *DREB2* specifically binds DRE element containing core sequence -CCGAC- that is involved in rapid expression of salt and drought stress responsive genes in *Arabidopsis* (Sakuma et al., 2006). bZIP proteins are well known for responding to ABA and stresses signaling. Several bZIP proteins can bind to ABRE (–ACGT–) elements and activate expression of ABRE-driven reporter genes, including *GmbZIP1* and *GmbZIP44* which are induced by drought, NaCl, cold, and ABA (Liao et al., 2008; Gao et al., 2011). Besides, *WRKY13, NAC11*, and *MYB84* are also involved in stress responses. Compared with controls, the expression profiles of stresses target genes have obvious differences in *GmANK114* transgenic hairy roots under drought and salt stresses, especially for *WRKY13, NAC11, DREB2, MYB84*, and *bZIP44* which were significantly up-regulated (Figure 12). This result suggest that *GmANK114* could change the expression of drought- and salt-related marker genes. That’s probably because *ANK114* indirectly affects the expression of these genes by affecting the function of other genes. However, it is not clear how *ANK114* affects the function of other genes to enhance stress resistance in soybean. Therefore, further studies are necessary to clarify the mechanism of the *GmANK114* participating in abiotic stresses.

## Conclusion

A total of 226 *ANK* genes were identified from the soybean genome. A member of the ANK-RF subfamily, *GmANK114*, improved tolerance against drought and salt stresses. This study provides important clues for future functional analysis of *ANK-RFs* in regulating of drought- and salt-related signal pathways in soybean.

## Data Availability Statement

The datasets presented in this study can be found in online repositories. The names of the repository/repositories and accession number(s) can be found in the article/Supplementary Material.

## Author Contributions

Z-SX and D-HM coordinated the project, conceived and designed the experiments, and edited the manuscript. J-YZ performed the experiments and wrote the first draft. Z-WL and YS conducted the bioinformatic work and performed the experiments. Z-WF, JC, Y-BZ, and MC contributed with valuable discussions. Y-ZM coordinated the project. All authors have read and agreed to the published version of the manuscript.

## Conflict of Interest

The authors declare that the research was conducted in the absence of any commercial or financial relationships that could be construed as a potential conflict of interest.

## Footnotes

1 http://pfam.sanger.ac.uk

2 https://phytozome.jgi.doe.gov/pz/portal.html

3 http://toolkit.tuebingen.mpg.de/blastclust

4 http://gsds.cbi.pku.edu.cn/

5 http://meme-suite.org/tools/meme

6 http://www.dna.affrc.go.jp/PLACE/

7 http://bioinformatics.psb.ugent.be/webtools/plantcare/html/

8 http://mg2c.iask.in/mg2c_v2.0/

9 http://chibba.pgml.uga.edu/mcscan2/

10 http://circos.ca/

11 http://www.blast2go.com

## Supplementary Material

Supplementary Figure 1 Gene structure analysis of the 226 *GmANK* genes. Untranslated regions (upstream/downstream) are displayed by blue box. Introns and exons are shown by black lines and yellow boxes, respectively. The lengths of introns and exons of each gene were displayed proportionally.

Supplementary Figure 2 Putative motifs of each soybean ANK proteins.

Supplementary Figure 3 Gene Ontology (GO) annotation for the GmANK proteins. BP: biological processes; MF: molecular functions; CC: cellular components.

Supplementary Figure 4 Sequence alignment of ANK-RF proteins in soybean.

Supplementary Figure 5 Germination rates of seeds in transgenic *Arabidopsis* under PEG6000 and NaCl treatments.

Supplementary Table 1 Description of soybean *ANK* genes.

Supplementary Table 2 FPKM values of different tissues of soybean *ANK-RF* genes.

Supplementary Table 3 FPKM values under drought, NaCl, and ABA treatment of soybean *ANK-RF* genes.

Supplementary Table 4 Primers used in this study.

Supplementary Table 5 Gene Ontology (GO) annotation for the GmANK proteins.
