# Supplementary figures and images for: The Ankyrin-Repeat Gene GmANK114 Confers Drought and Salt Tolerance in Arabidopsis and Soybean
Source: Front Plant Sci. 2020 Oct 29;11:584167. doi: 10.3389/fpls.2020.584167 (PMC7658197; doi:10.3389/fpls.2020.584167)

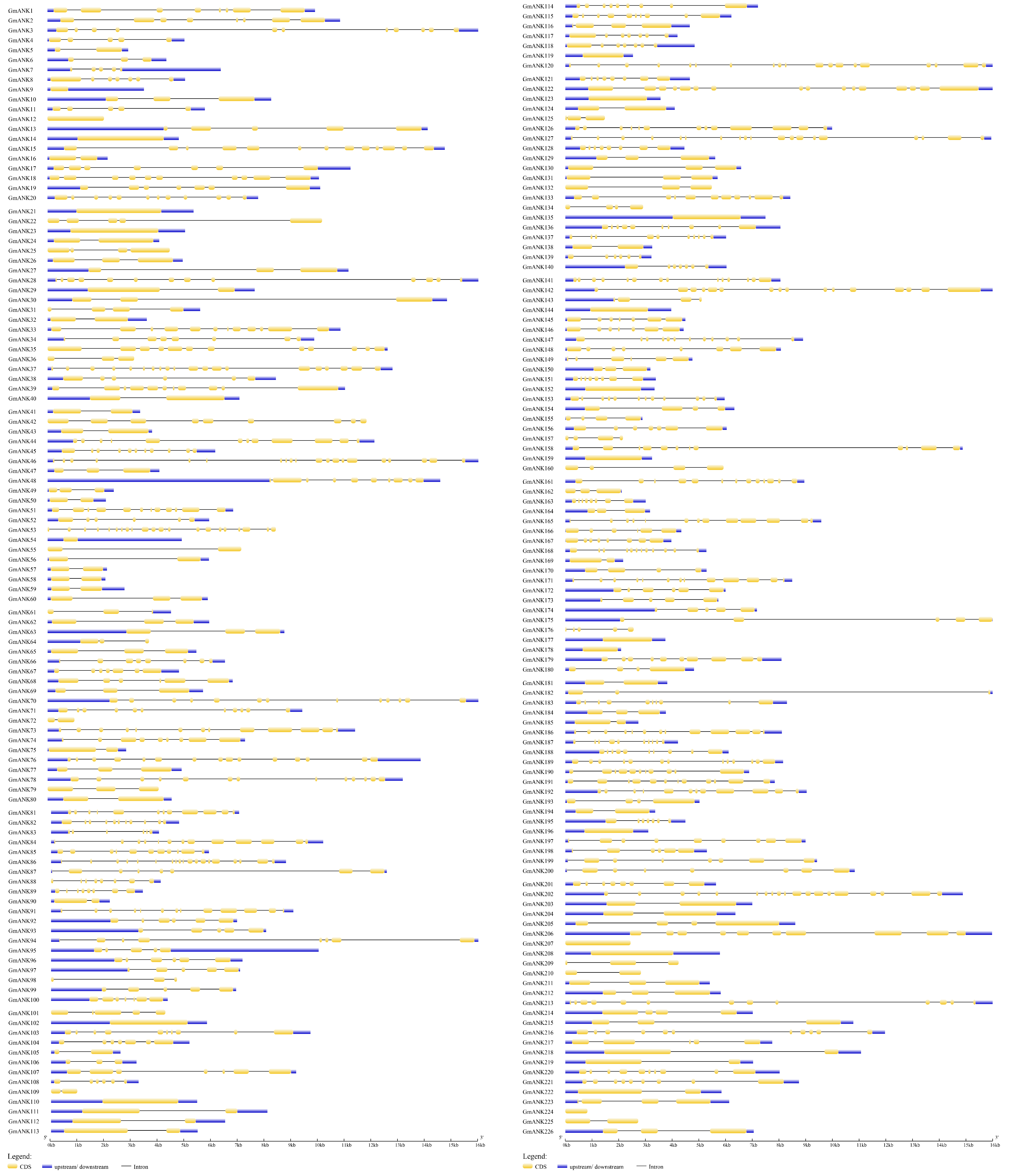

Supplement: Supplementary Figure 1 — Gene structure analysis of the 226 GmANK genes. Untranslated regions (upstream/downstream) are displayed by blue box. Introns and exons are shown by black lines and yellow boxes, respectively. The lengths of introns and exons of each gene were displayed proportionally. [file Image_1.JPEG]

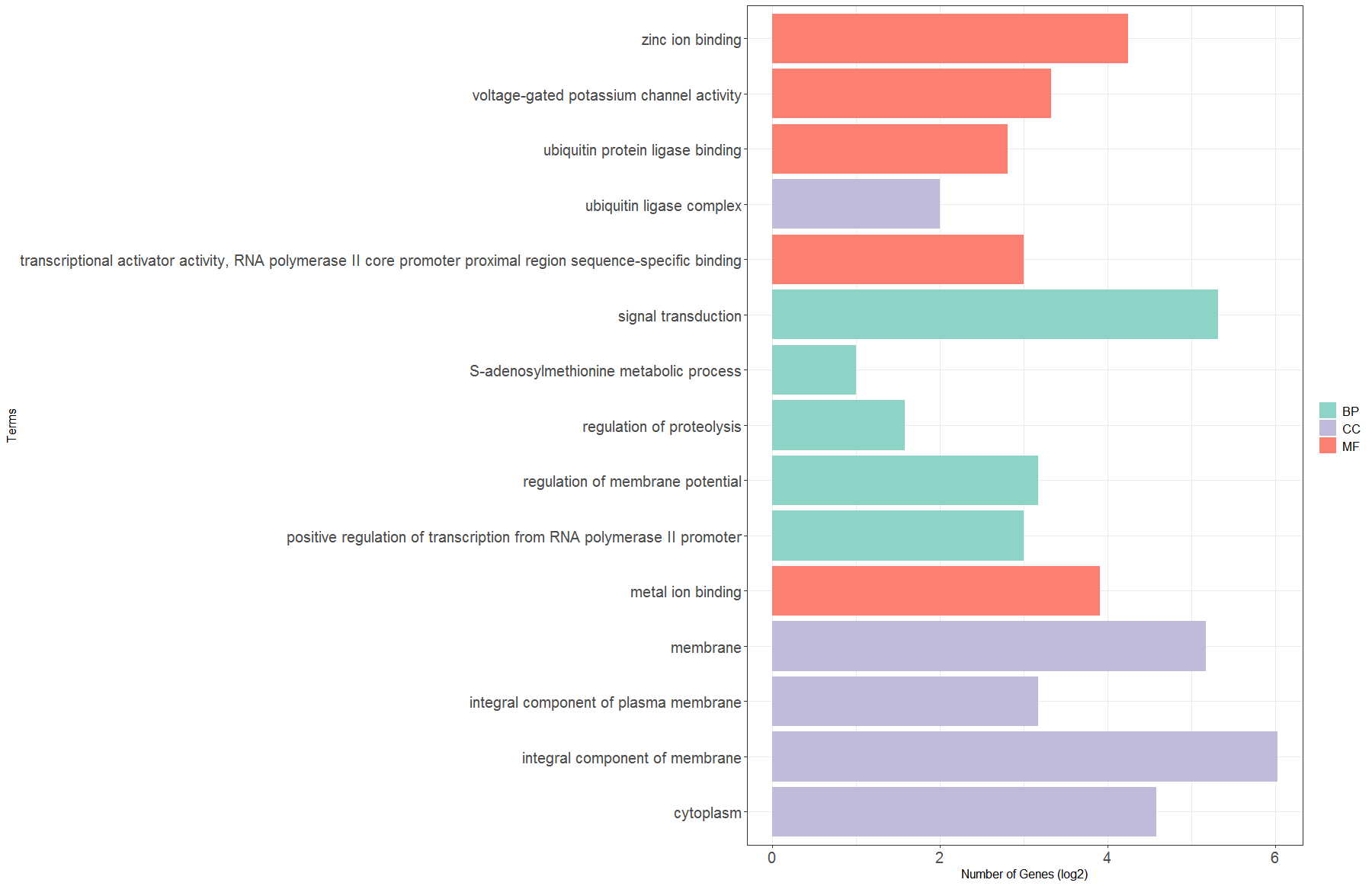

Supplement: Supplementary Figure 3 — Gene Ontology (GO) annotation for the GmANK proteins. BP: biological processes; MF: molecular functions; CC: cellular components. [file Image_3.TIFF]

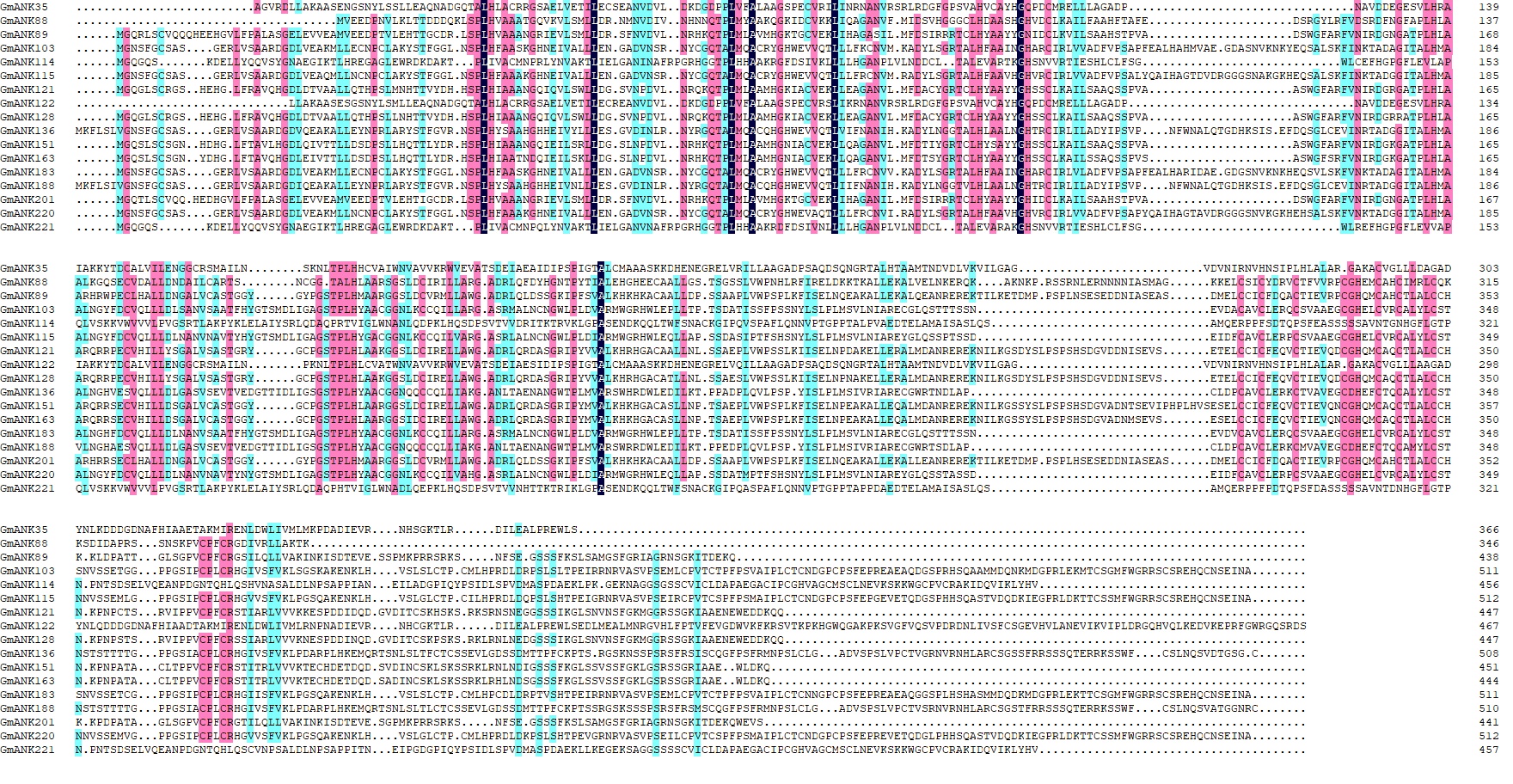

Supplement: Supplementary Figure 4 — Sequence alignment of ANK-RF proteins in soybean. [file Image_4.JPEG]

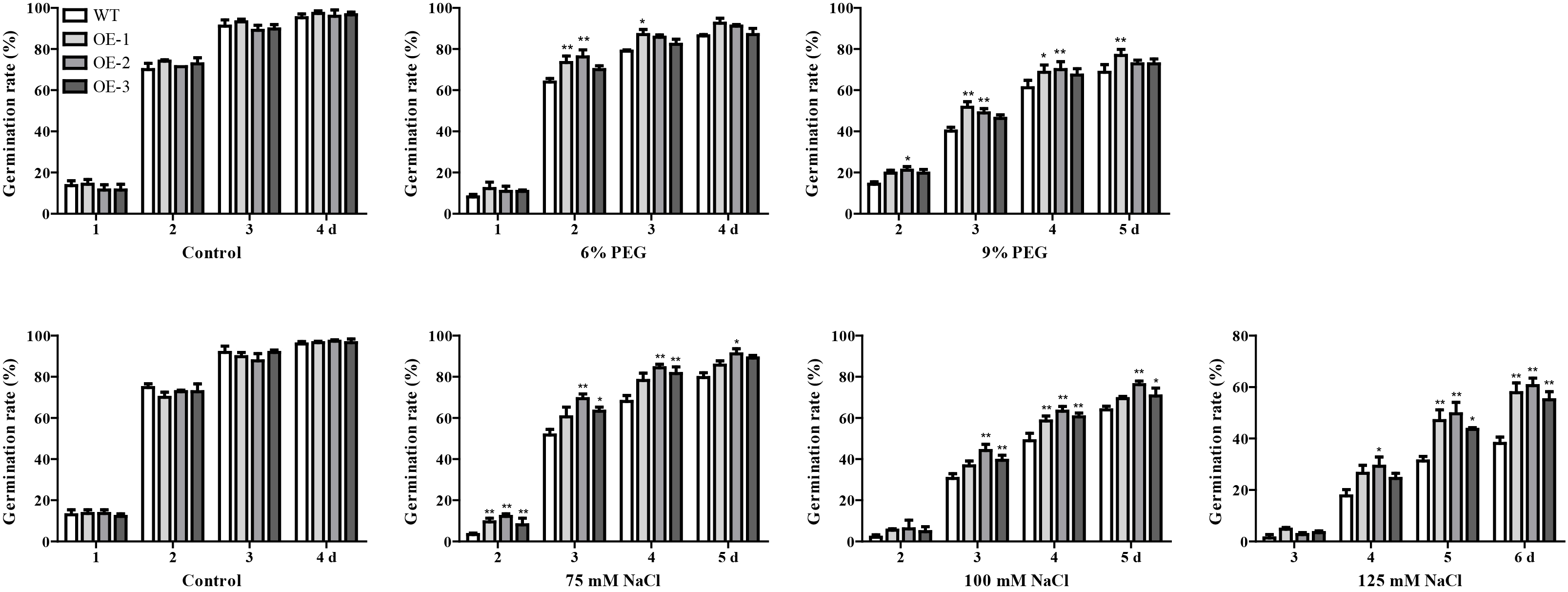

Supplement: Supplementary Figure 5 — Germination rates of seeds in transgenic Arabidopsis under PEG6000 and NaCl treatments. [file Image_5.JPEG]
